# Editorial: Plant genomics and pathogenomics: from technology to application in improving crop disease resistance

**DOI:** 10.3389/fpls.2024.1349113

**Published:** 2024-02-12

**Authors:** Vinay Panwar, Guus Bakkeren

**Affiliations:** ^1^ Department of Genetics and Plant Breeding, Chaudhary Charan Singh University, Meerut, India; ^2^ Agriculture and Agri-Food Canada, Summerland, BC, Canada

**Keywords:** plant genomics, pathogenomics, resistance genes, crop disease management, food security

The sustainable management of plant diseases is challenged by the world’s increasing demand for safe and diversified food during unprecedented global climate changes coupled with an increased incidence and severity of plant diseases due to altering pathogen evolution and their interaction with host plants. The emergence of new aggressive races of plant pathogens, intensification of abiotic stresses, groundwater resource depletion, and shrinking arable land are some of the major challenges that pose a great threat to future food security. Genome-enabled integration of research in the “post-genomic” era has refined our understanding of the complex interplay of dynamic molecular and cellular events and biochemical networks between partners in plant pathosystems. In the new millennium, we have progressed from sequencing single genomes to genome comparisons and generating highly refined pan-genome data for defining “core” and race-specific genes constituting weaknesses in rapidly evolving phytopathogens to be potentially exploited. For example, contextualized prediction and functional analysis of effector proteins (virulence factors) that contribute to pathogenicity and interact with host resistance genes carry practical ramifications for resistance durability and future resistance engineering (Upadhyaya et al., 2021). More recently, genomics research combined with revolutionary genome editing approaches has created new opportunities to explore fundamental aspects of biology in developing new technologies for offering high levels of protection against important diseases in cultivated crops.

In this Research Topic, we have collected original research and review articles to showcase the spectrum of the current efforts undertaken by applying modern genomics tools for crop improvement. The progress made in plant genome sequencing provides an opportunity that was not possible a decade ago to explore datasets to recruit new genes in various plant species. Such genome-wide studies have helped in the identification of gene members, gene structures, expression patterns, gene ontologies and evolutionary relationships, and protein interactions and to predict putative gene functions playing regulatory roles in plant–pathogen interactions (Demirjian et al., 2023). A genome-wide analysis of peach (*Prunus persica* L.) by Li et al. reports an alteration (upregulation) in the transcript level of members of the terpene synthase (*TPS*) gene family, involved in terpenoids biosynthesis, after inoculation with the fungus *Botryosphaeria dothidea*, a causative agent of peach gummosis. The work unravels a suite of *TPSs* that potentially contribute to the biosynthesis of linalool, a monoterpene resin volatile, known to play a role in plant defense. Correlation analysis demonstrates that *PpTPS* expression stimulates the release of linalool, suggesting that *TPSs* and/or their metabolites could potentially be involved in the defense response of peach plants against invading pathogens.

An impressive number of studies in recent years have demonstrated the feasibility of RNA interference (RNAi)-mediated transgenic and non-transgenic approaches such as virus-induced gene silencing (VIGS), host-induced gene silencing (HIGS), and spray-induced gene silencing (SIGS) for improving resistance against a wide array of plant pathogens in a variety of crops (Panwar et al., 2016; Panwar et al., 2017). These technologies rely mainly on silencing targeted genes in pests or pathogens by expressing the engineered dsRNA or small RNAs (siRNA/miRNA) in host plants. Exploiting a VIGS-based RNAi approach, Ibanez et al. show that down-regulation of the cathepsin B and Vitellogenin A1-like genes in the Asian citrus psyllid (ACP) *Diaphorina citri*, through in planta expression of homologous dsRNA, compromises the growth and reproductive capability of the insect feeding on RNAi-producing citrus plants. Such RNAi-based biocontrol strategies can provide a safe, versatile, and ecofriendly alternative to crop protection. Lately, VIGS-based silencing has gradually advanced into other areas as well, such as virus-mediated protein overexpression (VOX) and for *in vivo* delivery of genome editing components (virus-induced genome editing; VIGE) in plants (Rössner et al., 2022).

Wheat rust diseases caused by three rust fungi (leaf, stem, and stripe rust) belonging to the genus *Puccinia* are a major challenge to sustainable wheat production in the face of projected climate change. Breeding for rust disease resistance in wheat has been a strenuous exercise due to the continuous emergence and selection of evolving rust fungal races with new virulence to previously effective resistance (*R*) genes. Two review articles by Mapuranga et al. and Jost et al. included in this Research Topic provide a critical overview of the advances made in genomics and sequencing approaches to better understand the genetics and nature of rust pathogens and how this information can be explored for mining novel alleles/genes for expanding the arsenal of effective resistance sources to combat the devastating rust diseases. These articles highlight that prediction from secretomes of novel effectors with diverse characteristics using machine learning approaches and their in-depth functional characterization could provide valuable information on the evolutionary principles driving rust fungus evolution. Similarly, the identification, cloning, and study of unexploited *R* genes can increase our understanding of plant innate immunity beyond classic effectoromics. Once more, *R* genes are available, and their thoughtful stacking promises to be a strategy for durable rust disease control.

The evaluation of plant resistance against various pathogens plays a crucial role in disease management as this information is required for assessing which pathogen can infect and lead to disease. It helps decide what strategic and tactical control measures should be adopted in a timely manner. The article by Salotti et al. focuses on defining resistance patterns under field settings for 16 commercially grown grape varieties with respect to four major pathogens, namely, *Plasmopara viticola, Erysiphe necator, Botrytis cinerea*, and *Phyllosticta ampelicida*, which are responsible for causing downy mildew, powdery mildew, grey mold, and black rot disease, respectively, thus affecting grape production worldwide. Developing such decision control systems is crucial in plant disease risk assessment and integrated pest management.

In summary, this Research Topic comprises a diverse collection of both original research and review articles. The genetic and molecular studies presented in this Research Topic will help in advancing our current understanding of the cross-talk between host plants and their pathogens and the missing links involved in plant response to biotic stresses. They provide an outline for developing sustainable strategies for the prevention and control of economically important plant diseases. The knowledge generated through the published articles will help expedite crop improvement, thus playing an important role in increasing crop production for future food security.

## Author contributions

VP: Conceptualization, Formal analysis, Investigation, Visualization, Writing – original draft, Writing – review & editing. GB: Writing – review & editing.

## References

[B1] Demirjian C. Vailleau F. Berthomé R. Roux F. (2023). Genome-wide association studies in plant pathosystems: success or failure? Trends Plant Sci. 28 (4), 471–485. doi: 10.1016/j.tplants.2022.11.006 36522258

[B2] Panwar V. Jordan M. Mccallum B. Bakkeren G. (2017). Host-induced silencing of essential genes in *Puccinia triticina* through transgenic expression of RNAi sequences reduces severity of leaf rust infection in wheat. Plant Biotech. J. 16, 1013–1023. doi: 10.1111/pbi.12845 PMC5902777 28941315

[B3] Panwar V. McCallum B. Jordan M. Loewen M. Fobert P. McCartney C.. (2016). RNA silencing approaches for identifying pathogenicity and virulence elements towards engineering crop resistance to plant pathogenic fungi. CAB. Rev. 11:27. doi: 10.1079/PAVSNNR201611027

[B4] Rössner C. Lotz D. Becker A. (2022). VIGS goes viral: how VIGS transforms our understanding of plant science. Ann. Rev. Plant Biol. 73, 703–728. doi: 10.1146/annurev-arplant-102820-020542 35138878

[B5] Upadhyaya N. M. Mago R. Panwar V. Hewitt T. Luo M. Sperschneider J.. (2021). Genomics accelerated isolation of a new stem rust avirulence gene–wheat resistance gene pair. Nat. Plants 7, 1220–1228. doi: 10.1038/s41477-021-00971-5 34294906

